# Olive Actual “on Year” Yield Forecast Tool Based on the Tree Canopy Geometry Using UAS Imagery

**DOI:** 10.3390/s17081743

**Published:** 2017-07-30

**Authors:** Rafael R. Sola-Guirado, Francisco J. Castillo-Ruiz, Francisco Jiménez-Jiménez, Gregorio L. Blanco-Roldan, Sergio Castro-Garcia, Jesus A. Gil-Ribes

**Affiliations:** Department of Rural Engineering, University of Cordoba, E.T.S.I. Agronomos y Montes, Campus de Rabanales, Ctra. Nacional IV Km 396, 14014 Cordoba, Spain; g62caruf@uco.es (F.J.C.-R.); francisjimenez2@gmail.com (F.J.-J.); ir3blrog@uco.es (G.L.B.-R.); scastro@uco.es (S.C.-G.); gilribes@uco.es (J.A.G.-R.)

**Keywords:** olive, production forecast, manual canopy volume, individual crown area, tree mapping

## Abstract

Olive has a notable importance in countries of Mediterranean basin and its profitability depends on several factors such as actual yield, production cost or product price. Actual “on year” Yield (AY) is production (kg tree^−1^) in “on years”, and this research attempts to relate it with geometrical parameters of the tree canopy. Regression equation to forecast AY based on manual canopy volume was determined based on data acquired from different orchard categories and cultivars during different harvesting seasons in southern Spain. Orthoimages were acquired with unmanned aerial systems (UAS) imagery calculating individual crown for relating to canopy volume and AY. Yield levels did not vary between orchard categories; however, it did between irrigated orchards (7000–17,000 kg ha^−1^) and rainfed ones (4000–7000 kg ha^−1^). After that, manual canopy volume was related with the individual crown area of trees that were calculated by orthoimages acquired with UAS imagery. Finally, AY was forecasted using both manual canopy volume and individual tree crown area as main factors for olive productivity. AY forecast only by using individual crown area made it possible to get a simple and cheap forecast tool for a wide range of olive orchards. Finally, the acquired information was introduced in a thematic map describing spatial AY variability obtained from orthoimage analysis that may be a powerful tool for farmers, insurance systems, market forecasts or to detect agronomical problems.

## 1. Introduction

The olive crop covers more than 10 Mha in the world and has a notable social and economic importance in countries of Mediterranean basin, such as Spain that constitutes 44% and 22% of the global olive oil and table olive production, respectively [1]. Olive orchard profitability is highly influenced by yield which depends on orchard category [2] as well as harvesting season, due to alternate bearing [3]. This fact leads to discern between “on” and “off” years. Actual “on year” Yield (AY) is the potential yield in a forecasting model, so the yield actually achieved considering all limiting factors, and it is smaller than potential yield [4]. Thus, AY is the tree productivity, in kg tree^−1^, obtained in an “on year” and orchard actual “on year” Yield (OAY) is the orchard yield, in kg ha^−1^.

AY depends on several factors, such as environmental limitations, plant material or intercepted radiation, which is determined by canopy volume, leaf area index and leaf area density. The relationship between production and the canopy volume of a tree has been studied [5]. Tools to forecast AY would be highly useful to facilitate olive orchard management, policy making or to predict supply chain behavior, improving price stability.

It is possible to measure the canopy volume of trees using manual measurements or electronic devices [6]. Nonetheless, most applications in olive growing operations do not require accurate information of canopy volume because of the fact that manual canopy volume measurement with surveying rod is widely used as one goal of looking at overall tree shape and using a minimum number of measurements. Furthermore, aerial imagery from unmanned aerial systems (UASs) is a very promising method to characterize olive tree canopies [7]. It can be hypothesized that individual crown area obtained from an orthoimage may be highly correlated with canopy volume [8] assuming that, within an orchard, tree heights are rather uniform. Then, individual contour area of trees would be a useful parameter for other application that traditionally had been used, the tree canopy volume eliminating the dimension of tree height. The use of a 2D approach by ortho-mosaics would allow for the creation of site-specific management maps that could be related with geographic information systems (GIS) given an extra value. Furthermore, for farmers it is easier to use individual crown area than “apparent canopy volume” calculated by digital surface and terrain models; requiring less technical knowledge of aerial imagery with UAS combined with a simple image processing would be a valid tool to get individual crown area from the orthoimages generated, faster and easier than calculate canopy volume manually or digitally with point cloud.

The objective of this research was to assess tools for olive AY forecast based on tree canopy measurements for a wide range of olive orchards in southern Spain. AY was correlated with manual canopy volume. To provide a more useful method, we propose relating the manual canopy volume of trees to their individual crown area calculated from orthoimages acquired by UASs. This allowed for the creation of maps which could predict the AY for a particular use in a specific farm and enforcing the results understandability.

## 2. Material and Methods

### 2.1. Measurement and Relationship between AY and Manual Canopy Volume

Ten orchards were randomly selected from a research database among the most common orchard categories and layout [2]. Orchards are classified attending two variables: Irrigation (irrigated/rainfed) and orchard category (traditional/intensive/large hedgerow). Trees from the main olive cultivars in south Spain were included, such as Picual, Hojiblanca, Arbequina and Manzanilla. Super high-density orchards (more than 800 trees ha^−1^) were not considered in this study, considering that canopy volume profiles may be rectangular, truncated rectangular, or triangular [9] instead of ellipsoid volume assumed for the rest of orchard categories.

**Figure 1:**
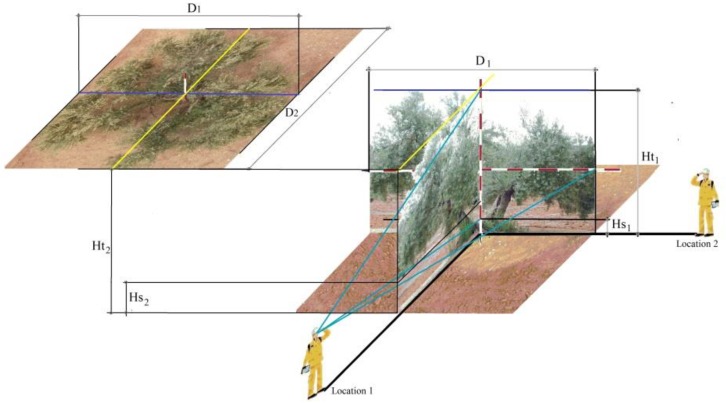
Manual Canopy Volume (MCV) measurement procedure for an olive tree.

The manual canopy volume of 518 trees located in different orchards was measured. The trees were harvested in four different harvesting seasons of “on years from 2011 to 2014” and fruits were weighed to obtain AY (kg tree^−1^). Manual canopy volume (MCV) in m^3^ was calculated following Equation (1) [10].

(1) $$\mathrm{MCV}\;=\frac{1}{6}{\pi \;D}_{1}\times D_{2}\times \frac{1}{2}\;(\left({\mathrm{Ht}}_{1}-{\mathrm{Hs}}_{1}\right)+\left({\mathrm{Ht}}_{2}-{\mathrm{Hs}}_{2}\right)$$

where D_1_ and D_2_ are crown diameters, Ht is tree height, Hs is skirt height, as the lowest canopy height from the ground. Heights were measured in two perpendicular positions. To obtain the diameters and heights of canopy geometry, two operators using a surveying rod were required using the next procedure (Figure 1): Firstly, operator A placed a surveying rod vertically at the olive canopy center. Then, operator B was located 10 m away from the surveying rod and took measurements of the outer canopy parts (Ht_1_ and Hs_1_). Afterward, operator A placed the surveying rod horizontally 1.5 m above the ground. The rod was extended from the two furthest points of the crown in that direction while operator B took the measurement of the canopy width (D_1_). Finally, these steps were performed again in a position located at a 90-degree angle from the first position.

A linear regression was described between manual canopy volume and AY. Two linear regressions were built separating data from irrigated orchards and rainfed ones. OAY, in kg ha^−1^ was calculated multiplying AY by planting density, in trees ha^−1^. Multiple regression models were avoided considering that AY forecasting tool was targeted to be used by farmers.

### 2.2. Measurement and Relationship between Manual Canopy Volume and Individual Crown Area

**Figure 2:**
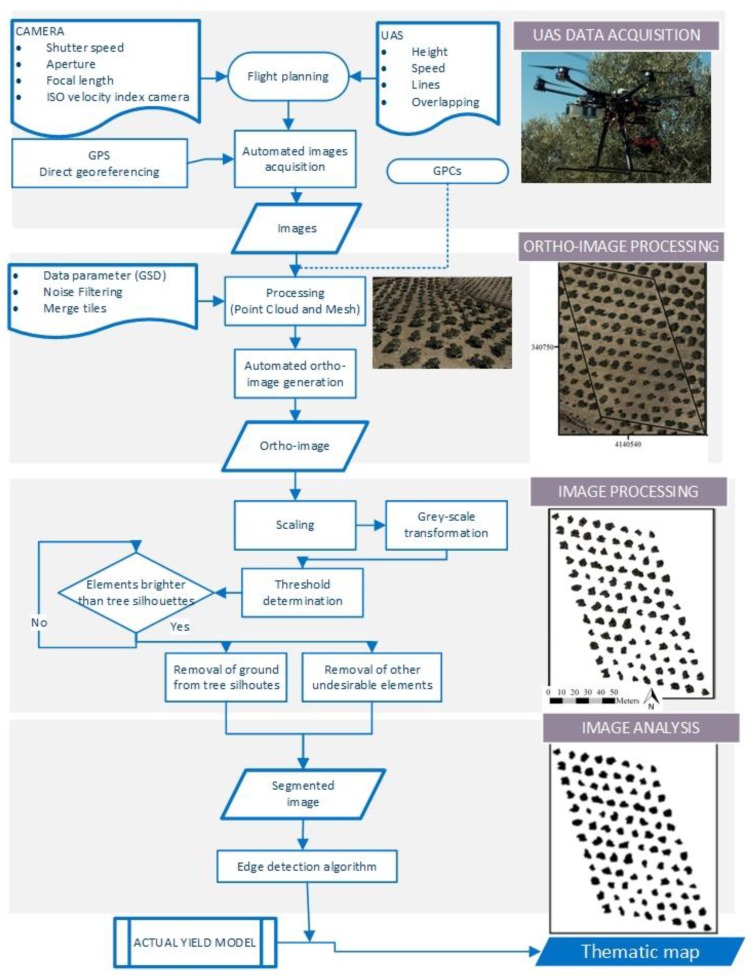
Orthoimage analysis procedure in a traditional olive orchard. UAS (Unmanned Aerial System), GPS (Global Positioning System), GSD (Ground Sample Distance), GPC (Ground Point Control).

A total of 36 trees from the most representative orchards studied (irrigated intensive, irrigated traditional and rainfed traditional) [1], were randomly selected (supplementary material). The trees were measured manually to determine manual canopy volume per tree (Figure 1), and digitally using the information acquired from a UAS to determine individual crown area (Figure 2). A linear regression was obtained to set a relationship between both variables.

Individual crown area was obtained from orthoimages acquired using a UAS. The UAS operated fully autonomously using waypoint navigation guidance for automatic image acquisition. The flight plan was set at 90 m height, 8 m s^−1^ cruising speed, and images were overlapped 85% and 70% in longitudinal and transversal directions respectively. Imagery was synchronized using a global positioning system (GPS) and triggering time was recorded for each image. There was no user interaction required for processing the acquired images during the flight. The pictures obtained from the UAS enabled orchard orthoimages creation using Pix4D (Ecublens). The matching image was calculated using the options “capture time”, “triangulation of image geolocation”, and “image similarity”. An older map of the area was used to set the ground control points. Aerial pictures were acquired using a digital camera (NEX7, Sony) with a shutter speed of 1:4000 s, an aperture of 3.617, a focal length of 18 mm and an ISO velocity index of 100, without a flash on windless days close to 12:00 p.m. The camera was placed in a gimbal (Zenmuse Z15, Dji) that maintained the camera position using 3 axes that were programmed using the aerial position. This device was fixed to a UAS frame (S800, Dji) controlled using a multirotor autopilot system (Wookong-M, Dji).

Orthoimages analysis (Figure 2) was performed using the open-source Java-based ImageJ package (ImageJ, National Institutes of Health). In the first stage, the image size was adjusted to the set measurement scale to convert pixel^2^ into m^2^ using a known measurement reference in the original image. Secondly, the original red, green, blue (RGB) values of the image were transformed into monochromatic grey-scale according to algorithm based on the lightness [11]. The green band was used to perform the segmentation procedure of the tree crown from the ground, based on the discrimination of undesirable elements using the RGB values of the individual pixels [12]. Manual segmentation of the image was made by applying a threshold as a function of the intensity values of the RGB image between 0 and 255 and labeling each pixel as black or white, depending on whether the pixel value was greater or less than the threshold selected. Two thresholds were selected, one to remove the ground from the image and another to remove the tree shadow. Once the tree canopy was separated by this binary process, the tree crown area was calculated using the automated routine tool known as “analyze particles” based on edge detection algorithms, which numbered and outlined each tree [13].

Finally, AY was estimated with individual crown area by the relation between individual crown area and manual canopy volume. A plot of one orchard was mapped for predicting AY with the relationship obtained by using the individual crown area measured.

## 3. Results and Discussion

### 3.1. Orchard Actual “on Year” Yield and Other Features

Olive yield was influenced by many factors such as canopy dimensions, orchard layout, water availability or other stresses. Farmers are aware of these complex relations. However, all these variables are often summed up in orchard yield as the main factor for orchard profitability. Within the same orchard category, OAY increased when the planting density increased, while AY exhibited the opposite trend (Table 1). Fruit set and fruit fresh weight may influence AY [14] along with planting density. The ratio between production and canopy volume (kg m^−3^) was generally higher for denser plantations, ranging from 1.6 to 3.1 kg m^−3^ for orchards with 70 trees ha^−1^ and 408 trees ha^−1^, respectively, though there were exceptions due to specific orchard constraints. Other authors describe lower production efficiency ratios, i.e., 1 to 0.26 kg m^−3^, depending on the tree cultivar [15].

The mean OAY in the irrigated orchards was higher than that in the rainfed ones. The mean OAY in the irrigated large hedgerows (12,172 kg ha^−1^) was slightly higher than that for irrigated traditional orchards (11,241 kg ha^−1^) and irrigated intensive orchards (10,640 kg ha^−1^). By contrast, the rainfed orchards had much lower mean “on year” yields, from 6496 kg ha^−1^ produced by the traditional orchards to 5729 kg ha^−1^ for intensive orchards. Yield values were in accordance with those described in Spain for new plantations (aged 3 to 7 years old) with 408 trees ha^−1^, which produced 9540 kg ha^−1^, and with 816 trees ha^−1^, which produced 13,898 kg ha^−1^ [10]. Previous research also stated that the mean yield was influenced by tree training, planting density and location, with yields ranging between 6380 kg ha^−1^ and 10,580 kg ha^−1^ [3].

It was notable that high OAY values could be obtained in any irrigated orchard category with any tree training system. The lack of differences indicated that the profitability of orchard categories is not due only to their OAY, but other limiting factors, such as operational cost, inputs requirements, orchard size, or lower harvesting machinery performance. In this way, the number of high-density and hedgerow orchards is increasing, although traditional orchards are still the most widely used category of the total cultivated olive area. The linear regression (Equation (2)) predicted actual “on year” yield depending on manual canopy volume for a wide range of olive orchard categories (Table 1).

### 3.2. Actual “on Year” Yield Forecasting Tool Based on Manual Canopy Volume

A general regression (Equation (2)) to predict actual “on year” yield (AY) in kg per tree depending on manual canopy volume (MCV) was built for both irrigated and non-irrigated orchards. The regression showed a highly linear trend (r^2^ = 0.76, *p* ≤ 0.01) and a standard error of 18.6 kg tree^−1^ for the mean estimation. The provided forecast equation was representative for south Spain considering the wide range of trees, orchard categories, locations and harvesting seasons tested. Further research is needed to adjust the model coefficients for other locations with different soil, climate constraints, or cultivars [16]. All data represented the yields from “on years”, considering that production in “off year” did not provide a significant regression to describe tree production according to manual canopy volume (data not shown). The general regression showed the relationship between tree size and yield which have been described relating olive tree productivity to trunk girth [17]. This trend has also been proven in other crops, such as apple, in which orchard yield and photosynthetically active radiation were correlated with tree training system or tree architecture, canopy volume and the trunk cross-sectional area [18].

AY = 15.928 + 1.215 × MCV(2)

The general regression can be separated into two different specific forecast equations, one for irrigated and one for rainfed orchards.

(3) Irrigated:AY=10.642+1.541×MCV

(4) Rainfed:AY=25.932+0.781×MCV

**Figure 3:**
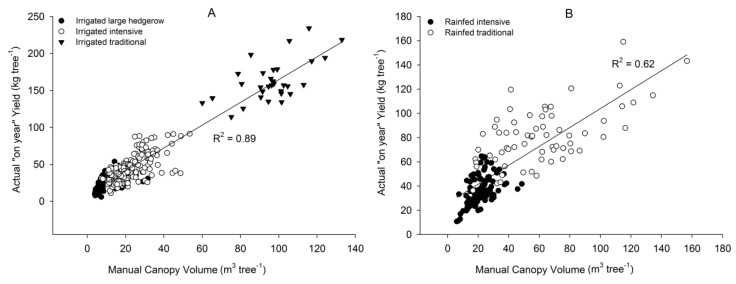
Linear model between the Manual Canopy Volume and the Actual “on year” Yield (AY) for the irrigated orchards (**A**) and for the rainfed orchards (**B**).

Linear regression was obtained for irrigated orchards (r^2^ = 0.89, *p* ≤ 0.01) and 13.1 kg tree^−1^ as the standard error of the mean estimated (Figure 2, Equation (3)), whereas the regression for the rainfed orchards showed a slightly worse adjustment (r^2^ = 0.62, *p* ≤ 0.01), and 17.1 kg tree^−1^ as the standard error of the mean estimate (Equation (4)) (Figure 3).

Irrigation improved AY by reducing water-limiting factor [4], although AY in rainfed orchards was close to that obtained in the irrigated orchards in rainy years. Accordingly, the soil variability resulted in higher data scattering for the rainfed orchards, while the irrigated orchards showed a scatter pattern that was similar to the general regression. These results agreed with previous research showing that olive production is strongly influenced by irrigation, although the response gradually decreases when the water applied approaches the maximum demand [19].

Not only canopy volume per tree has a significant influence on AY, but the canopy volume per hectare determined OAY, which should be maximized in order to increase crop profitability. It is advisable to adapt pruning intensity to reach optimal orchard canopy volume [10], considering that tree density, canopy size and soil management are strategic decisions for olive water relations [20] and then, for olive AY. For high-density orchards under climate conditions in southern Spain, the optimal canopy volume should be around 8000 m^3^ ha^−1^ for rainfed orchards, while it should be between 11,000 and 13,000 m^3^ ha^−1^ for irrigated ones [19]. For all studied orchards, the orchard canopy volume was below optimal values so it might be advisable to increase orchard canopy volume to increase OAY, although in some cases, vigorous trees may decrease harvesting performance reducing olive orchard profitability [21]. Similarly, it is important to choose when and how to perform the pruning according to the harvesting method to enhance the harvest effectiveness based on the branches’ vibrations [22].

### 3.3. Actual “on Year” Yield Forecasting Tool Based on Tree Crown Area

Manual canopy volume was an adequate predictor for AY (Figure 3), although the method introduces several errors. Firstly, it does not consider the high variability between crown shapes [23] and estimates the canopy volume assuming that all trees had an ellipsoid volume. Secondly, manual measurements might show a lower resolution than other available systems, because of the accuracy of the operator related to how well he can identify the crown contour. Furthermore, this method requires remarkable time consumption for taking measurements. In this work, the time spent for measuring 1 ha (96 trees) of a traditional orchard was approximately 4 h, with a mean value of 2.5 min tree^−1^. However, although the method may be tedious and have high labour requirements for large areas, it could be valid for smaller ones.

Significant differences (*p* < 0.05) for AY, manual canopy volume and individual tree crown area matched between orchard categories (Table 2). Intensive orchard had smaller canopy volume, and the irrigated orchard volumes were less scattered than the rainfed ones. Therefore, irrigation not only increases AY but also reduces orchard variability.

**Figure 4:**
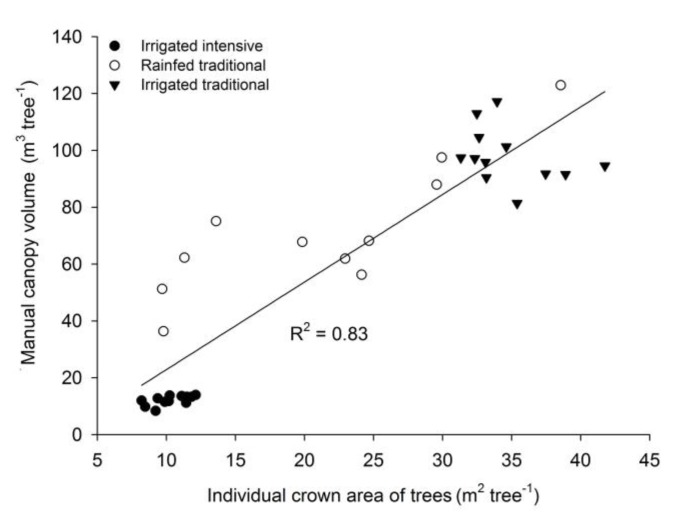
Linear model between the individual crown area (ICA) of trees and the manual canopy volume (MCV) for three different orchard categories.

A linear regression was built to determine the relationship between manual canopy volume (MCV) in m^3^ and the individual tree crown area (ICA) in m^2^ for different orchard categories (Figure 4) (r^2^ = 0.83, *p* ≤ 0.01) (Equation (5)) because tree height was rather similar in commercial orchards, which was 3.8 ± 0.2 m for the irrigated traditional orchards and 3.9 ± 0.4 m for the rainfed orchards (mean ± standard deviation). This relationship may simplify the process of measurements and tree characterization for this purpose and does not need to be carried out using complex methods. Results confirmed that it is possible to estimate canopy volume from orthoimagery obtained from a commercial digital camera mounted on an inexpensive UAS by applying Equation (5) which resulted in a standard error of the mean estimate of 16.77 m^3^ tree^−1^.

(5) MCV=−7.906+3.080×ICA

Canopy volume estimation from aerial imagery may include tree height measurements obtained from Digital Surface Models (DSMs), though this does require data processing that increases the computation time. However, for determining individual tree crown and density arrangements, this information is not necessary. UAS orthoimagery has been appropriated for determining the individual crown area of the trees, although this can be estimated by obtaining data from other aerial remote method. In this way, satellite or UAS imagery could be useful sources for estimating these parameters using very high-resolution satellites such as Pleiades, WorldView-2 or OrbView, which provide images with a resolution of up to 0.4 m that allows for the recognition, identification and delineation of individual tree crowns using object-based image analysis [24]. Digital elevation models using traditional airborne platforms can also be used for tree delineation [25], although the images obtained might make the process more expensive and unaffordable for small- and medium-sized orchards, considering that they are adapted to cover large areas. Therefore, UASs is a flexible tool for the acquisition of digital images to get thematic maps in any moment without depending on cloud condition.

The resolution of orthoimages obtained has been acceptable, having a resolution up 0.02 m, and, therefore, the procedure has supposed a promising alternative to characterize the canopy of olive trees [7]. These models describe the surfaces of both terrain and trees, so the detection and delineation of trees represents an effective technique for imagery analysis. The processing result demonstrates that the proposed method was not affected by the structure of the vegetation or by ground unevenness. Moreover, it was not necessary to use any radiometric normalization because the mosaic created did not comprise a large number of images collected in the same period of time under the same conditions. In this work, the threshold values for segmentation were set manually and the tree delineation was achieved automatically. Nonetheless, the assessment of individual crown area may be performed automatically using specific algorithms and image-processing software [26]. Further advances should accomplish a fully automated process.

**Figure 5:**
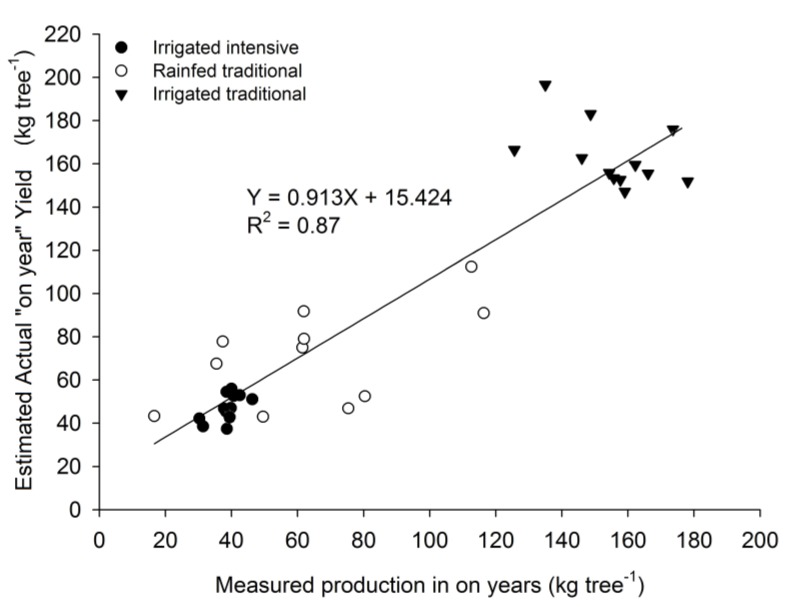
Predicted AY and measured production in an “on-year”.

Manual canopy volume was replaced by individual crown area in the specific regressions for irrigated and rainfed orchards (Equations (3) and (4)). Estimated value of AY using individual crown area was compared to measured yield, providing good adjustment slope close to 1:1 (Figure 5). These equations could be used in a wide range of olive orchard categories from large hedgerow orchards to traditional ones.

Further studies should include a real-time system to detect single trees and tree canopies measures might be integrated [27] into new agricultural machinery for different labour requirements, such as pest and disease control or to determine harvesting labour using specific mapping. It could be used to develop innovations for olive harvesting machinery; for instance, they could adapt vibration parameters (e.g., vibration power, frequency, amplitude) to tree canopy volume in order to enhance harvesting efficiency [28].

**Figure 6:**
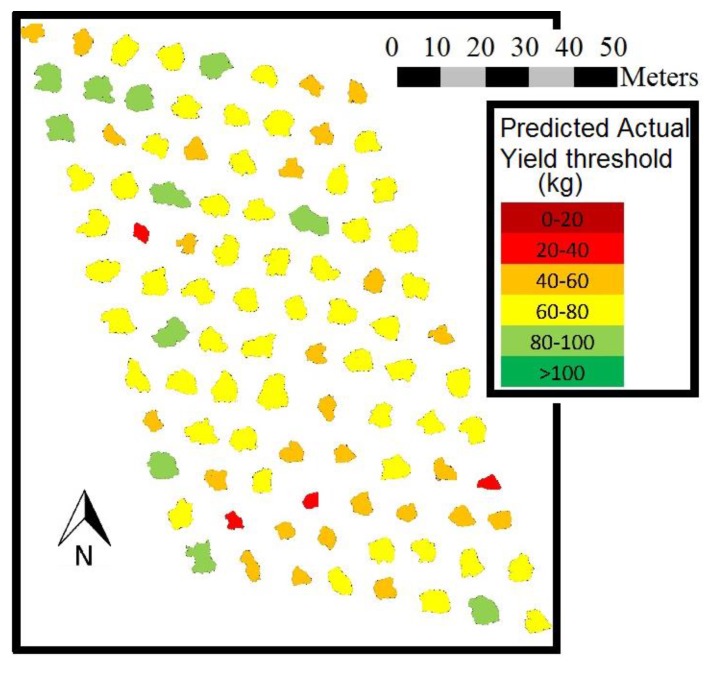
Mapping of the expected Actual Yield in a rainfed traditional olive orchard related to the variability in the individual crown area of the trees.

Orthoimages analysis may provide valuable information for farmers representing acquired information on thematic maps. A plot of 1 hectare of rainfed traditional olive trees was mapped using this technique (Figure 6). As a result, AY was calculated based on each tree individual crown area. Therefore, canopy volume or individual crown area mapping could be useful tools to describe spatial yield variability. The information provided by these maps should be integrated into precision agriculture decision-making processes to enhance orchard management efficiency.

## 4. Conclusions

This research provides new advances for the description of broadly applicable methodology to describe tree and orchard actual “on year” yield for both oil ant table olives and for all olive orchard categories except super high-density ones. Significant relationships were described between tree crown features and actual yield (AY), so forecasts for AY can be performed using manual canopy volume, but also by using individual crown area, simplifying the method to calculate the canopy volume over large areas, and avoiding other tree crown measurements. Once AY could be forecasted, OAY might be estimated for a farm, area, or even for a region using quick and inexpensive methodology. Nonetheless, these relationships are valid for southern Spain conditions, thus, further research is required to obtain the adjusted coefficients for other geographical areas. Irrigation was a key factor that should be considered to assess AY. However, there were no big differences in OAY between the studied orchard categories. An optimal canopy volume per hectare should be achieved to reduce yield gap by tree training, taking into account the enhancements in harvesting operations. Crown features measurements and yield forecasting provide very valuable maps for farmers, agricultural insurance systems and researchers to characterize an orchard, to enhance orchard management and to predict economic, agronomical or social aspects.

## Figures and Tables

**Table 1:** Orchards and trees parameters measured in “on year” season. Parameters are the mean ± standard deviation.

Orchard Category	Planting Density (tree ha^−1^) – (Planting Distance)	Tree Production or Actual Yield (kg tree^−1^)	Orchard Actual Yield (kg ha^−1^)	Manual Canopy Volume (m^3^ tree^−1^)	Production Per Canopy Volume (kg m^−3^)	Orchard Canopy Volume (m^3^ ha^−1^)
Irrigated large hedgerow	555 (6 × 3 m)	31.4 ± 9.3	17,463	11.7 ± 3.4	2.8 ± 0.8	6723
	408 (7 × 3.5 m)	24.2 ± 11.4	9883	7.6 ± 2.5	3.1 ± 0.9	3099
	312 (8 × 4 m)	29.3 ± 6.1	9171	21.6 ± 5.3	1.4 ± 0.3	6760
Irrigated intensive	285 (7 × 5 m)	53.3 ± 17.3	15,251	19.9 ± 9.5	2.9 ± 0.6	5690
	208 (6 × 8 m)	39.0 ± 16.8	7479	21.4 ± 9.3	1.9 ± 0.6	4655
	204 (7 × 7 m)	45.1 ± 15.4	9190	23.9 ± 8.1	1.9 ± 0.5	4879
Rainfed intensive	158 (7 × 9 m)	45.2 ± 11.5	7181	21.4 ± 5.1	2.2 ± 0.7	3395
	138 (8 × 9 m)	31.4 ± 9.6	4278	22.3 ± 9.6	1.5 ± 0.4	3023
Irrigated traditional	70 (12 × 12 m)	162.9 ± 27.9	11,241	96.4 ± 15.6	1.7 ± 0.3	6652
Rainfed traditional	70 (12 quincunx)	81.2 ± 23.6	6496	61.2 ± 30.6	1.6 ± 0.8	4893

**Table 2:** Tree parameters of common orchards measured in “on year” season used for AY estimation. Parameters are the mean ± standard deviation values. Different letters within a column show significant differences between orchard categories (Duncan’s post hoc test, *p* < 0.05).

Orchard Category	Production in or Actual Yield (kg tree^−1^)	Manual Canopy Volume (m^3^ tree^−1^)	Individual Crown Area (m^2^ tree^−1^)
Irrigated intensive	38.6 ± 4.3 a	12.1 ± 1.7 a	10.3 ± 1.3 a
Rainfed traditional	65.8 ± 29.8 b	73.6 ± 27.6 b	24.2 ± 13.4 b
Irrigated traditional	155.2 ± 15.0 c	98.0 ± 9.9 c	34.8 ± 3.1 c

## Supplementary Materials

Supplementary File 1
