# Supplementary material for: Olive Actual “on Year” Yield Forecast Tool Based on the Tree Canopy Geometry Using UAS Imagery
Source: Sensors (Basel). 2017 Jul 30;17(8):1743. doi: 10.3390/s17081743 (PMC5579829; doi:10.3390/s17081743)
Supplement: Supplementary File 1 [file sensors-17-01743-s001.pdf]

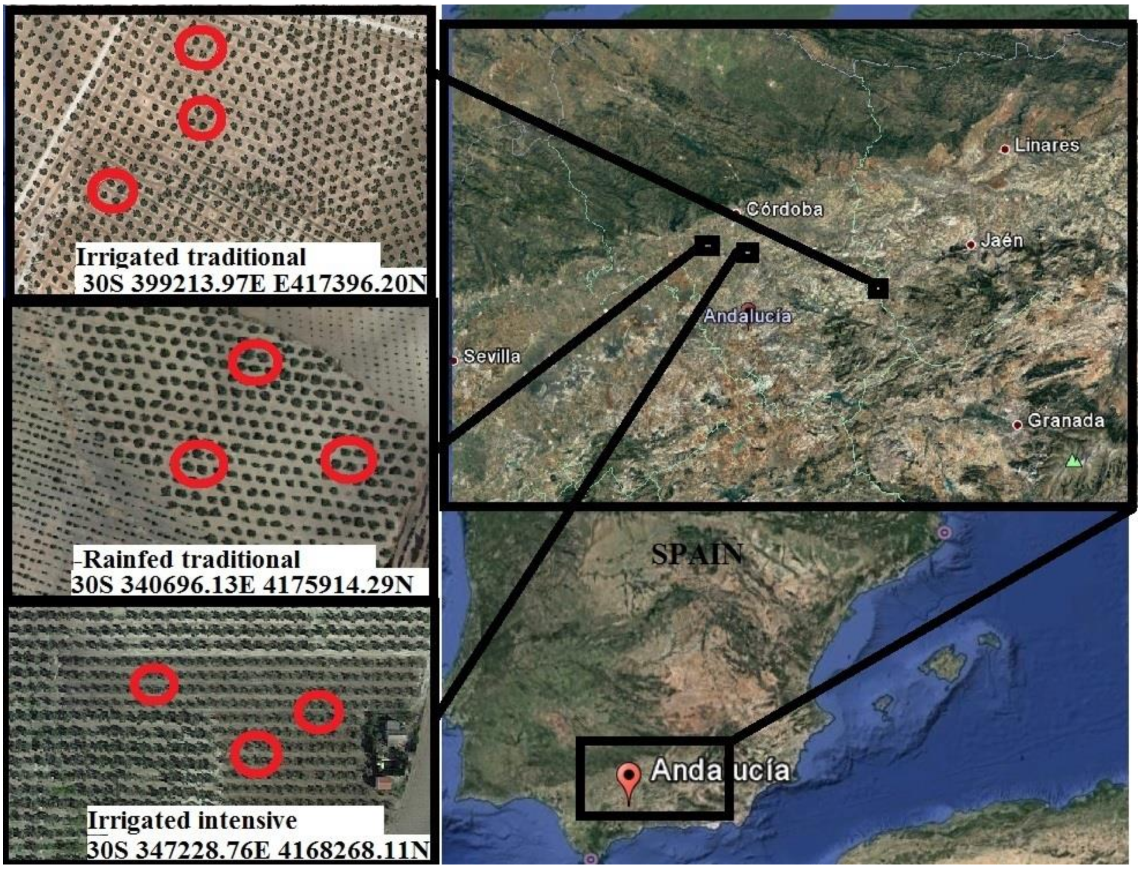

Figure 0. Geographical distribution of olive orchards and trees investigated for the model between the individual crown contour of trees (ICA) manual canopy volume (MCV) and actual yield (AY)
